# Diet cost plays a key role in determining the risk of pediatric attention deficit hyperactivity disorder: Findings from a case–control study

**DOI:** 10.1002/fsn3.3026

**Published:** 2022-08-09

**Authors:** Khadijeh Abbasi, Sahar Foshati, Sanaz Mehrabani, Reza Ghiasvand, Mohammad Bagherniya, Mohammad Hossein Rouhani

**Affiliations:** ^1^ Department of Community Nutrition, Food Security Research Center, School of Nutrition and Food Science Isfahan University of Medical Sciences Isfahan Iran; ^2^ Department of Clinical Nutrition, Food Security Research Center, School of Nutrition and Food Science Isfahan University of Medical Sciences Isfahan Iran

**Keywords:** attention deficit hyperactivity disorder, case–control studies, diet cost, diet quality

## Abstract

The aim of our study was to assess the association between diet cost and attention deficit hyperactivity disorder (ADHD) in children. This study was a case–control study conducted in Isfahan, Iran. Based on the Diagnostic and Statistical Manual of Mental Disorders‐V criteria, a total of 200 children aged 4–12 years with ADHD and 300 age‐ and sex‐matched children without ADHD, respectively, participated in case and control groups. A validated food frequency questionnaire that contained 168 food items was used to assess the dietary intake. The cost of food items was obtained from licensed markets. The food price was corrected for edible portion sizes as well as food weight changes due to cooking process. Our results indicated that diet cost per 1000 kcal was significantly lower in the case group compared with the control group (60,843.48 ± 6987.83 vs. 67,828.33 ± 8989.48 Rials, *p* < .01). In the crude model, a significantly lower risk of ADHD was observed in the higher quartiles of diet cost per 1000 kcal (odds ratio (OR) = 0.06; 95% confidence interval (CI) = 0.03, 0.13; *p* < .001). This finding remained significant, even after adjustment for potential confounders such as age, gender, body mass index (BMI), socioeconomic status (SES), and intakes of eicosapentaenoic acid (EPA), docosahexaenoic acid (DHA), and saturated fatty acids (SFA). Therefore, it seems that the risk of ADHD is inversely associated with diet cost in children. Further studies, particularly longitudinal ones, are warranted.

## Graphical abstract

Diet cost was lower in the case group than in the control group.An inverse association was found between diet cost and the risk of ADHD in children.

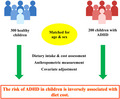

## 1 INTRODUCTION

Attention deficit hyperactivity disorder (ADHD) is one of the most prevalent neuropsychiatric diseases in children characterized by behavioral symptoms of impulsivity, hyperactivity, and inattention (Loe & Feldman, 2007). The global average prevalence of this childhood disorder is around 5%. In addition, a further 5% of children manifest the aforementioned behavioral symptoms, but these symptoms are just subthreshold to meet ADHD diagnostic criteria (Sayal et al., 2018). Accumulating evidence suggests that genetics and environmental factors, especially dietary intake, have important roles in the incidence of ADHD (Wolraich et al., 2011).

Some observational studies reported that sugar intake may increase the risk of ADHD (Lien et al., 2006). A significant dose–response association was even observed between the consumption of sugar‐sweetened beverages and the risk of ADHD (Millichap & Yee, 2012; Yu et al., 2016). These beverages are rich in additives, preservatives, and artificial food colors which may affect children's behavior (Millichap & Yee, 2012; Schnoll et al., 2003). Moreover, blood levels of docosahexaenoic acid (DHA) in patients with ADHD were reported to be lower than in healthy subjects (Antalis et al., 2006). It was shown that supplementation with omega‐3 fatty acid has a modest therapeutic effect on ADHD (Bloch & Qawasmi, 2011). Furthermore, it was reported that a healthy traditional dietary pattern is associated with lower odds of ADHD (Woo et al., 2014) and a Western dietary pattern including processed foods and snacks is associated with higher odds of ADHD (Howard et al., 2011; Yan et al., 2018).

Previous studies reported that higher diet quality is associated with lower emotional symptoms in patients suffering from ADHD (Kohlboeck et al., 2012). Diet quality is mainly influenced by diet cost (Monsivais et al., 2010). It seems that low‐cost diets have high energy density and low nutrient contents because they are rich in sweets, refined grains, and fats. In contrast, high‐cost diets have low energy density and high nutrient contents because they are rich in proteins, fibers, vitamins, and minerals. Therefore, many researchers suggested that high‐cost diets are healthier than low‐cost diets (Andrieu et al., 2006; Rao et al., 2013; Rehm et al., 2011). Interestingly, recent studies reported significant inverse associations between diet cost and the risk of obesity, insulin resistance, and type 2 diabetes mellitus (Conklin et al., 2016; Heilskov Rytter et al., 2015).

Diet cost may have a role in dietary choices and consequently the risk of some diseases. Nevertheless, to the best of our knowledge, no studies have evaluated the association between diet cost and the risk of ADHD. Therefore, we aimed to conduct a case–control study to investigate the association between diet cost and ADHD in children.

## 2 METHODS

### 2.1 Design and participants

A case–control study was conducted in Isfahan, Iran in 2017. Five hundred children aged 4–12 years participated in this study with a case–control ratio of 1:1.5 (200 cases and 300 controls). Cases were enrolled in the study from psychotherapy clinics located at different community areas of Isfahan (south, north, east, west, and center) using a random cluster multistage sampling method. Controls were also enrolled in the study from public health centers located at different community areas of Isfahan (south, north, east, west, and center) by the same sampling method. Cases were children with ADHD diagnosed by a psychiatrist according to the Diagnostic and Statistical Manual of Mental Disorders‐V criteria (American Psychiatric Association, 2013). Controls were healthy children with no history of chronic diseases. Particularly, they were screened for the absence of ADHD. Both case and control groups did not follow a specific diet plan or have certain food prohibitions. They were also matched for sex and age (maximum age difference was 6 months). Before the enrollment, a consent form was obtained from the participants and their parents. It is worth mentioning that the Ethics Committee of Isfahan University of Medical Sciences approved the study protocol (IR.MUI.REC.1395.3529).

### 2.2 Anthropometric measurements

Height was measured with 0.1 cm precision by means of a stadiometer (Seca, Hamburg, Germany) while the participants were standing upright against a stadiometer in bare feet. Weight was measured to the nearest 0.1 kg using a digital medical scale (Seca, Hamburg, Germany) while the participants were wearing light clothing without shoes. Body mass index (BMI) was calculated by dividing weight in kilograms by height in meters squared.

### 2.3 Dietary assessment

A semiquantitative food frequency questionnaire (FFQ) containing 168 food items was used to assess habitual dietary intake of the participants during the previous 1‐year period. The validity and reliability of this FFQ were acceptable (Mirmiran et al., 2010). It was completed by the parents of the participants. In order to eliminate seasonal variation in dietary intake, the dietary intake of both case and control groups was obtained in the same season. The mentioned FFQ had six choices (Never, 1–3 times/month, 1 time/week, 2–4 times/week, 5–6 times/week, and daily) to indicate the average frequency of intake. The reported frequency was then converted to daily intake. Household measures were also used to convert portion sizes to grams. Finally, the actual food intake (g/day) was analyzed using Nutritionist IV software modified for Iranian foods (version 7.0; N‐Squared Computing, Salem, OR, USA) to determine the amount of macro‐ and micronutrient intake.

### 2.4 Cost assessment

The price of each food item was obtained from licensed markets. The food price was corrected for edible portion sizes as well as food weight changes due to cooking process. Afterwards, the sum of all food item prices was considered as the total daily diet cost for each participant. Finally, diet cost was reported in Rials (the official Iranian currency) per 1000 kcal.

### 2.5 Covariates

Based on epidemiological studies, age and sex are related to ADHD. Therefore, cases and controls were matched for age (years) and sex (male or female). A general questionnaire including demographic and medical data was completed for each subject through a face‐to‐face interview. A self‐reported questionnaire including questions about housing tenure, international travel, owning a car, family size, and education and employment of household head was also administered to assess socioeconomic status (SES). According to SES, participants were categorized into three groups including low SES, middle SES, and high SES.

### 2.6 Statistical analysis

The independent‐samples *t* test was used to evaluate between‐group differences in age, height, BMI, and diet cost per 1000 kcal. The chi‐square test was used to assess between‐group differences in gender and SES. The multivariate analysis of variance (MANOVA) was used to determine energy‐adjusted nutrient intake across quartiles of diet cost per 1000 kcal in both cases and controls. Each nutrient intake was adjusted for energy intake by the residual method. Three models of the logistic regression analysis were used to examine the risk of ADHD across quartiles of diet cost per 1000 kcal. The first model was a crude model with no adjustment for confounders. The second model (model I) was adjusted for age, gender, BMI, and SES; and the third model (model II) was additionally adjusted for eicosapentaenoic acid (EPA), docosahexaenoic acid (DHA), and saturated fatty acids (SFA). A *p*‐value less than .05 was considered statistically significant. All statistical analyses were performed using Statistical Package for Social Sciences version 18 (SPSS Inc., Chicago, IL, USA).

## 3 RESULTS

All enrolled children completed the study successfully (*n* = 500). Baseline characteristics of the participants are presented in Table 1. There were no significant differences in age (*p* = .36) and gender (*p* = .49) between the two groups. Nevertheless, the SES of the subjects was significantly different between the two groups (*p* = .049). Participants with low SES were significantly more in the case group than in the control group. Moreover, diet cost per 1000 kcal was significantly lower in the case group compared with the control group (60,843.48 ± 6987.83 vs. 67,828.33 ± 8989.48 Rials, *p* < .01). Furthermore, healthy subjects were likely to be taller (*p* = .003) and had higher BMI (*p* = .001) than subjects with ADHD.

**TABLE 1:** General characteristics of cases and controls^a^

Characteristics	Case (*n* = 200)	Control (*n* = 300)	*p*‐Value^b^
Age (year)	7.07 ± 1.71	6.93 ± 1.66	.36
Gender			
Male	79.5	76.9	.496
Female	20.5	23.1	
Height (cm)	120.86 ± 0.77	123.62 ± 10.5	.003*
Body mass index (kg/m^2^)	13.71 ± 2.49	14.60 ± 3.16	.001*
Socioeconomic status			
Low	54	29.2	.049*
Middle	30	32.6	
High	16	38.3	
Diet cost per 1000 kcal (Rials)	60,843.48 ± 6987.83	67,828.33 ± 8989.48	<.001*

^a^ Values were expressed as mean ± standard deviation or percentage.

^b^ The independent‐samples *t* test and the chi‐square test were used for comparison of quantitative and qualitative data, respectively.

* Between‐group differences were significant (*p* < .05).

Energy‐adjusted nutrient intake of both groups across quartiles of diet cost per 1000 kcal is shown in Table 2. Dietary intake of protein, fiber, zinc, vitamin A, vitamin B1, vitamin B6, vitamin B12, and vitamin C was significantly higher in the last quartile of diet cost per 1000 kcal compared with the first quartile (*p* < .001 for all). In contrast, participants in the top quartile of diet cost per 1000 kcal had a significantly lower intake of carbohydrate, fat, and SFA (*p* < .001 for all).

**TABLE 2:** Dietary intake of nutrients across quartiles of diet cost in Rials per 1000 kcal in both cases and controls^a^

Nutrients	Q1	Q2	Q3	Q4	*p*‐Value^b^
	<60,000 (*n* = 125)	60,001–63,000 (*n* = 125)	63,001–69,000 (*n* = 125)	69,000< (*n* = 125)	
Carbohydrate (g)	110.98 ± 13.15	108.69 ± 12.42	107.05 ± 9.24	105.25 ± 10.32	.001*
Protein (g)	14.26 ± 15.80	5.61 ± 16.41	23.53 ± 16.24	36.7 ± 8.75	<.001*
Fat (g)	38.03 ± 8.43	42.12 ± 8.1	36.05 ± 7.32	31.73 ± 4.68	<.001*
Saturated fatty acids (g)	10.97 ± 2.5	11.76 ± 2.90	9.26 ± 2.25	7.93 ± 1.57	<.001*
Fiber (mg)	3.27 ± 5.50	0.78 ± 5.88	4.73 ± 4.02	7.94 ± 2.30	<.001*
Zinc (mg)	1.52 ± 1.49	0.80 ± 1.41	2.32 ± 1.49	3.35 ± 0.68	<.001*
Vitamin A (mcg)	303.33 ± 87.65	167.44 ± 394.57	345.36 ± 283.84	533.42 ± 316.81	<.001*
Vitamin B1 (mg)	1.48 ± 0.26	1.58 ± 0.22	1.68 ± 0.30	1.96 ± 0.17	<.001*
Vitamin B6 (mg)	0.42 ± 0.31	0.29 ± 0.39	0.56 ± 0.21	0.72 ± 0.10	<.001*
Vitamin B12 (mcg)	1.39 ± 0.69	2.79 ± 1.92	2.81 ± 1.66	2.71 ± 0.84	<.001*
Vitamin C (mg)	23.56 ± 50.84	7.15 ± 60.78	45.30 ± 40.24	68.23 ± 28.07	<.001*

^a^ Values were expressed as mean ± standard deviation and adjusted for energy intake by the residual method.

^b^ *p*‐Values were obtained using the multivariate analysis of variance (MANOVA).

* The trends were significant (*p* < .05).

The risk of ADHD across quartiles of diet cost per 1000 kcal is presented in Table 3. In the crude model, a significantly lower risk of ADHD was observed in higher diet cost per 1000 kcal (odds ratio (OR) = 0.06; 95% confidence interval (CI) = 0.03, 0.13; *p* < .001). Likewise, the highest quartile of diet cost per 1000 kcal was significantly related to a reduced risk of ADHD after adjustment for potential confounders in model I (OR = 0.03; 95% CI = 0.01, 0.09; *p* < .001) and model II (OR = 0.04; 95% CI = 0.01, 0.09; *p* < .001).

**TABLE 3:** Odds ratio (OR) (95% confidence interval (CI)) of attention deficit hyperactivity disorder (ADHD) across quartiles of diet cost in Rials per 1000 kcal

Models	Q1	Q2	Q3	Q4	*p*‐Value^a^
	<60,000 (*n* = 125)	60,001–63,000 (*n* = 125)	63,001–69,000 (*n* = 125)	69,000< (*n* = 125)	
Crude model	1 (ref)	1.54 (0.93, 2.55)	0.49 (0.29, 0.81)	0.06 (0.03, 0.13)	<.001*
Model I^b^	1 (ref)	1.34 (0.75, 2.41)	0.38 (0.21, 0.67)	0.03 (0.01, 0.09)	<.001*
Model II^c^	1 (ref)	1.27 (0.71, 2.29)	0.40 (0.22, 0.71)	0.04 (0.01, 0.09)	<.001*

^a^ *p*‐Values were obtained using the logistic regression analysis.

^b^ Model I was adjusted for age, gender, body mass index (BMI), and socioeconomic status (SES).

^c^ Model II was additionally adjusted for eicosapentaenoic acid (EPA), docosahexaenoic acid (DHA), and saturated fatty acids (SFA).

* The trends were significant (*p* < .05).

## 4 DISCUSSION

Food price is one of the main determinants of people's food choices, especially in low‐ and middle‐income countries such as Iran. To the best of our knowledge, the present study is the first one to investigate the association between diet cost and the risk of ADHD. Interestingly, our results indicated that the risk of ADHD is significantly associated with diet cost in children. In addition, our results showed that diet cost is a significant indicator of nutrient density.

In our study, the risk of ADHD was decreased with increasing diet cost. ADHD is a neurodevelopmental disorder. Therefore, one possible explanation for the aforementioned finding is that diet cost determines the dietary intake of nutrients which are essential for neurodevelopment in children. As our results demonstrated, dietary intake of protein, fiber, zinc, vitamin A, vitamin B1, vitamin B6, vitamin B12, and vitamin C was increased with increasing diet cost. In contrast, dietary intake of carbohydrate, fat, and SFA was decreased with increasing diet cost. In previous studies, it was also shown that protein intake and fiber intake have a positive and direct correlation with diet cost, while carbohydrate intake and SFA intake have a reverse correlation with diet cost (Andrieu et al., 2006; Rehm et al., 2011). In addition, several previous studies have declared that the intake of nutrients, such as zinc, iron, magnesium, vitamin A, vitamin B1, vitamin B6, vitamin B12, vitamin C, and fiber, can reversely contribute to the risk of ADHD (Del‐Ponte et al., 2019).

Although the exact etiology of ADHD is not discovered, some evidence indicated that abnormal metabolism of amino acids, vitamins, and minerals has an important role in the pathogenesis of ADHD (Sharma & Couture, 2014). Particularly, it was reported that children suffering from ADHD are usually deficient in zinc as well as vitamin B6 (Greenblatt & Delane, 2017). Zinc is essential for normal brain development and function, some metal–enzyme complexes, and conversion of vitamin B6 to its active form. The active form of vitamin B6, pyridoxal phosphate, is needed to make dopamine, noradrenalin, gamma‐aminobutyric acid, and serotonin (Heilskov Rytter et al., 2015; Kohlboeck et al., 2012). It is worth mentioning that meat as an expensive food item in Iran is a rich source of zinc and vitamin B6. Therefore, it is not surprising that the intake of these two neuro‐nutrients had significant increasing trends across quartiles of diet cost per 1000 kcal in our study.

Based on previous studies, it was suggested that the risk of ADHD increases with increasing dietary intake of carbohydrate, especially glucose (Millichap & Yee, 2012; Yu et al., 2016). In our study, dietary intake of carbohydrate was highest in the first quartile of diet cost and lowest in the last quartile of diet cost, and diet cost was inversely associated with the risk of ADHD. Therefore, it seems that our findings are in line with the previous literature. Children with ADHD have impulsive behaviors that lead them to unhealthy food habits and choices such as refined cereals, pastries, and soft drinks. These foods have low prices, few nutrients, and high contents of carbohydrates (Lien et al., 2006). Refined rice and bread are two commonly consumed examples of these kinds of foods in Iran.

According to our results, it seems that expensive diets are healthier than inexpensive ones. This finding seems to be reasonable because healthy foods are usually nutrient‐dense, and nutrient‐dense foods tend to cost more than foods that have high energy content but minimal nutritional value (Drewnowski, 2010). Nevertheless, following a healthy low‐cost diet is not impossible. It is achievable by changing government nutrition policies and adding some new dietary suggestions. For instance, providing rebates and price subsidies for several healthy foods and substituting animal proteins such as meats with plant proteins such as legumes and soybeans may reduce diet cost while preserving nutritional quality.

As a side note, rising food prices have a more negative effect on the consumption of healthy foods than unhealthy foods, particularly in low‐ and middle‐income countries. For example, a 10% increase in the price of soft drinks leads to an 8%–10% reduction in their consumption, while a 10% increase in the price of fruits, vegetables, and dairy products results in a 70% reduction in their consumption (Green et al., 2013). Therefore, rising food prices can indirectly replace healthy foods with unhealthy ones and consequently may increase the risk of ADHD. This is a key point which should be considered in government nutrition policies, especially during economic crises.

Three limitations of our study are needed to be acknowledged. First, a cause‐and‐effect relationship cannot be determined in our study. Second, we mainly collected data by self‐reported questionnaires which are subject to recall and estimation biases. Third, we did not differentiate between subtypes of ADHD. Apart from these limitations, the strength of this study is that we did our best to control confounding factors. We designed this research as an age‐ and sex‐matched case–control study. In addition, we adjusted for well‐known potential confounders in the statistical analysis.

In conclusion, our findings suggest that the risk of ADHD is inversely associated with diet cost in children. Due to the point that diet cost is greatly influenced by diet quality, our findings can shed light on the importance of diet quality for the prevention of ADHD in children. Food and nutrition policymakers should take these findings into account, though longitudinal studies are required to determine the casual relationship.

## FUNDING INFORMATION

This work was financially supported by the Isfahan University of Medical Sciences [grant number 395529].

## CONFLICT OF INTEREST

The authors declare that there is no conflict of interest.

## ETHICS STATEMENT

The Ethics Committee of Isfahan University of Medical Sciences approved the study protocol (IR.MUI.REC.1395.3529).

## Data Availability

The data that support the findings of this study are available from the corresponding author, upon reasonable request.
